# Obituary

**Published:** 1863-05

**Authors:** 


### OBITUARY.

DIED—In Angelica, Allegany County, April 24th, 1863, of erysipelas RICHARD CHARLES, M. D., aged 65 years and 5 months.

Dr. Charles was born in the County of Tyrone, Ireland; he received his medical education at Dublin, Glasgow and New York; he emigrated to America in 1821, and commenced the practice of his profession in the town of Angelica in 1825, where he continued to reside until the time of his death. As a medical practitioner Dr. Charles occupied a high and prominent position. His sound sense, his acknowledged skill as a physician, his uncompromising hostility to all forms of quackery, his sterling integrity as a man, his genial disposition and gentle manners will long continue to be held in grateful remembrance.

